# Interaction effect between NAFLD severity and high carbohydrate diet on gut microbiome alteration and hepatic *de novo* lipogenesis

**DOI:** 10.1080/19490976.2022.2078612

**Published:** 2022-05-29

**Authors:** Hyena Kang, Hyun Ju You, Giljae Lee, Seung Hyun Lee, Taekyung Yoo, Murim Choi, Sae Kyung Joo, Jeong Hwan Park, Mee Soo Chang, Dong Hyeon Lee, Won Kim, GwangPyo Ko, Innovative Target Exploration of NAFLD (ITEN) consortium

**Affiliations:** a Department of Environmental Health Sciences, Graduate School of Public Health, Seoul National University, Republic of Korea; b Center for Human and Environmental Microbiome, Institute of Health and Environment, Seoul National University, Republic of Korea; c Bio-MAX/N-Bio, Seoul National University, Seoul, Republic of Korea; d Department of Biomedical Sciences, Seoul National University College of Medicine, Seoul, Republic of Korea; e Division of Gastroenterology and Hepatology, Department of Internal Medicine, Seoul National University College of Medicine, Seoul Metropolitan Government Boramae Medical Center, Seoul, Republic of Korea; f Department of Pathology, Seoul National University College of Medicine, Seoul Metropolitan Government Boramae Medical Center, Seoul, Republic of Korea; g KoBioLabs Inc, Seoul, Republic of Korea

**Keywords:** Nonalcoholic fatty liver disease, gut microbiome, high carbohydrate diet, *de novo* lipogenesis, liver transcriptome

## Abstract

Nonalcoholic fatty liver disease (NAFLD) is associated with high carbohydrate (HC) intake. We investigated whether the relationship between carbohydrate intake and NAFLD is mediated by interactions between gut microbial modulation, impaired insulin response, and hepatic *de novo* lipogenesis (DNL). Stool samples were collected from 204 Korean subjects with biopsy-proven NAFLD (n = 129) and without NAFLD (n = 75). The gut microbiome profiles were analyzed using 16S rRNA amplicon sequencing. Study subjects were grouped by the NAFLD activity score (NAS) and percentage energy intake from dietary carbohydrate. Hepatic DNL-related transcripts were also analyzed (n = 90). Data from the Korean healthy twin cohort (n = 682), a large sample of individuals without NAFLD, were used for comparison and validation. A HC diet rather than a low carbohydrate diet was associated with the altered gut microbiome diversity according to the NAS. Unlike individuals from the twin cohort without NAFLD, the abundances of *Enterobacteriaceae* and *Ruminococcaceae* were significantly different among the NAS subgroups in NAFLD subjects who consumed an HC diet. The addition of these two microbial families, along with *Veillonellaceae*, significantly improved the diagnostic performance of the predictive model, which was based on the body mass index, age, and sex to predict nonalcoholic steatohepatitis in the HC group. In the HC group, two crucial regulators of DNL (*SIRT1* and *SREBF2*) were differentially expressed among the NAS subgroups. In particular, kernel causality analysis revealed a causal effect of the abundance of *Enterobacteriaceae* on *SREBF2* upregulation and of the surrogate markers of insulin resistance on NAFLD activity in the HC group. Consuming an HC diet is associated with alteration in the gut microbiome, impaired glucose homeostasis, and upregulation of hepatic DNL genes, altogether contributing to NAFLD pathogenesis.

## Graphical abstract

**Figure:**
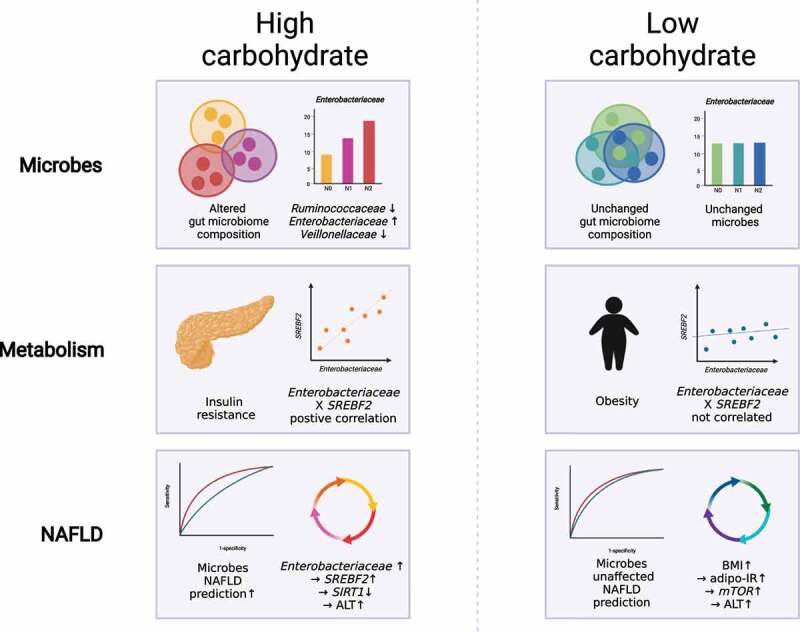

## Introduction

Nonalcoholic fatty liver disease (NAFLD) is associated with metabolic syndrome, obesity, and diabetes mellitus (DM)^1^. In addition, NAFLD has genetic and dietary risk factors.^2^ Patients with nonalcoholic steatohepatitis (NASH) and advanced fibrosis present more adverse clinical outcomes,^3^ and should be potentially targeted for pharmacotherapy. However, in NAFLD patients, lifestyle modification is primarily pursued as a cost-effective intervention to control metabolic dysfunction.^4^ As a result, further investigation of the underlying mechanisms by which dietary patterns affect NAFLD progression are merited to elucidate its complex pathogenesis.

Sources of lipid influx contributing to NAFLD development include lipid biosynthesis, mostly from carbohydrate (through *de novo* lipogenesis [DNL]^5^), free fatty acids (FFAs) derived from lipolysis of adipose tissue,^6^ and excessive dietary fat in the form of chylomicrons absorbed from the intestine.^7^ Among them, DNL contributes up to 26% of the total triglycerides (TG) synthesis in NAFLD patients with hyperinsulinemia, while serum-derived non-esterified fatty acids account for approximately 59% and the diet account for 15%;^8^ the rate of DNL is three times higher in NAFLD patients than in body mass index (BMI)-matched healthy controls.^5^ Circulating glucose and insulin levels can also induce hepatic DNL in NAFLD patients.^9^ Thus, elucidating the contribution of carbohydrates, the primary source of DNL, to NAFLD progression is crucial for optimizing effective dietary interventions that ameliorate NAFLD.

A growing body of evidence suggests that altered gut microbial ecology is associated with the development and progression of NAFLD.^10–12^ Specifically, the diversity and composition of the gut microbiome are significantly different in non-obese NAFLD subjects, resulting in the depletion of *Ruminococcaceae* and the enrichment of *Veillonellaceae*.^11^ Changes in the gut microbiome are closely linked with host-microbe-nutrient interactions, such as dietary patterns; in turn, dietary patterns are associated with NAFLD progression.^13,14^ Indeed, a 2-week dietary intervention involving an extremely carbohydrate-restricted diet (4% of total energy intake from carbohydrate) improved fat metabolism in the liver by modulating the folate-producing gut microbiome.^15^ A 4-week dietary intervention that increased fiber intake also led to the modulation of gut microbiome-mediated glucose homeostasis in an overweight population.^16^ Despite the evident interaction among macronutrients, the gut microbiome, and NAFLD, further mechanistic studies that investigate these relationships in humans are needed.

In this study, we attempted to elucidate the effect of dietary patterns on NAFLD pathogenesis using a multi omics approach in a well-characterized biopsy-proven NAFLD cohort. We aimed to provide new insights into the pathomechanism of NAFLD as related to dietary carbohydrate patterns, alterations of the gut microbiome, and hepatic DNL.

## Results

### Study population characteristics

This study included 129 Korean individuals with biopsy-proven NAFLD (NAFL, n = 70; NASH, n = 59) and 75 controls without NAFLD (Supplementary Table 1). Subjects were divided into two groups according to their carbohydrate intake (high carbohydrate [HC] group, energy intake from carbohydrates ≥70%; low carbohydrate [LC] group, energy intake from carbohydrates <70%).^17,18^ Baseline characteristics did not significantly differ between the HC and LC groups (Table 1). Subjects were then subdivided into three subgroups (N0–2) according to their NAFLD activity score (NAS). The subgroups were categorized as non-NASH (N0), borderline NASH (N1), and definite NASH (N2) (Table 1 and Supplementary Table 2).

**Table 1.** Clinical characteristics of all study subjects stratified by carbohydrate intake and the NAFLD activity score

	HC (n = 107)				LC (n = 97)				
NAFLD cohort	Non NASH	Borderline NASH	Definite NASH	P value	Non NASH	Borderline NASH	Definite NASH	P value	P value (HC vs. LC)
Clinical characteristics									
No. of subjects (%)	38 (36)	37 (35)	32 (30)		37 (38)	33 (34)	27 (28)		
Age (years)	56.7 ± 13.5	54.2 ± 13.9	49.1 ± 14.8	0.138	57.7 ± 8.9	46.0 ± 16.9	46.2 ± 12.1	<0.001***	0.127
Male (%)	21 (55)	14 (38)	11 (34)	0.156	16 (43)	20 (61)	11 (41)	0.248	0.482
BMI (kg/m^2^)	25.3 ± 3.8	27.3 ± 3.8	27.4 ± 3.9	0.021*	25.2 ± 2.8	28.9 ± 4.0	29.0 ± 3.7	<0.001***	0.138
AST (IU/L)	30.2 ± 19.2	41.2 ± 24.8	71.2 ± 75.5	<0.001***	25.3 ± 8.8	62.6 ± 62.6	61.8 ± 41.4	<0.001***	0.389
ALT (IU/L)	33.6 ± 29.0	53.4 ± 36.5	92.3 ± 80.7	<0.001***	28.1 ± 15.1	76.6 ± 49.8	100.9 ± 72.9	<0.001***	0.324
GGT (IU/L)	35.9 ± 37.1	47.2 ± 67.7	54.5 ± 41.1	<0.001***	46.5 ± 51.9	62.6 ± 42.1	65.2 ± 38.9	0.008**	0.003**
Cholesterol (mg/dL)	178.8 ± 40.7	190.4 ± 45.8	182.2 ± 32.7	0.583	185.3 ± 38.2	183.3 ± 38.6	200.8 ± 36.2	0.079	0.217
HDL (mg/dL)	49.1 ± 11.9	46.5 ± 9.2	46.8 ± 11.2	0.764	48.5 ± 12.0	48.8 ± 10.6	44.7 ± 10.5	0.374	0.942
LDL (mg/dL)	101.7 ± 34.8	105.3 ± 35.3	107.9 ± 28.6	0.718	106.7 ± 32.5	107.3 ± 42.6	131.5 ± 23.2	0.034*	0.096
TG (mg/dL)	127.8 ± 69.8	180.4 ± 127.3	148.2 ± 67.6	0.174	128.0 ± 65.3	139.7 ± 50.8	158.5 ± 50.9	0.009**	0.841
FFA (µEq/L)	583.3 ± 222.7	636.5 ± 247.8	691.3 ± 255.1	0.074	597.7 ± 223.6	681.7 ± 294.4	657.5 ± 242.2	0.484	0.968
TB (mg/dL)	2.27 ± 9.45	0.87 ± 0.4	0.64 ± 0.19	0.010*	0.80 ± 0.38	0.77 ± 0.27	0.87 ± 0.37	0.431	0.357
Alb (g/dL)	4.09 ± 0.29	4.18 ± 0.31	4.24 ± 0.24	0.135	4.09 ± 0.26	4.27 ± 0.27	4.31 ± 0.29	0.002**	0.510
Platelet (x10^3^/µL)	227.3 ± 60.1	237.1 ± 53.9	244.3 ± 56.6	0.500	241.7 ± 47.5	260.3 ± 62.2	241.5 ± 40.9	0.617	0.162
C-peptide (ng/mL)	2.61 ± 1.49	3.83 ± 2.59	4.14 ± 4.68	0.002**	2.52 ± 0.97	4.49 ± 3.57	4.24 ± 2.14	<0.001***	0.156
hs-CRP (ng/mL)	0.18 ± 0.49	0.20 ± 0.31	0.24 ± 0.38	0.018*	0.17 ± 0.32	0.19 ± 0.15	0.31 ± 0.32	<0.001***	0.130
Ferritin (ng/mL)	116.1 ± 71.5	126.6 ± 79.8	249.6 ± 330.7	0.108	105.3 ± 56.2	263.3 ± 311.7	228.0 ± 146.6	<0.001***	0.054
HA (ng/mL)	44.8 ± 36.5	56.8 ± 59.0	41.5 ± 32.5	0.484	53.2 ± 68.9	62.6 ± 104.2	43.4 ± 29.5	0.487	0.560
HbA1c (%)	5.96 ± 1.06	6.03 ± 0.63	6.29 ± 0.87	0.031*	5.96 ± 0.63	6.09 ± 0.93	5.98 ± 0.46	0.832	0.799
FBS (mg/dL)	109.2 ± 27.6	113.9 ± 31.3	114.6 ± 30.2	0.071	109.2 ± 27.6	113.9 ± 31.3	114.6 ± 30.2	0.561	0.672
Insulin (µIU/mL)	12.9 ± 10.1	15.6 ± 7.46	16.9 ± 8.01	0.005**	11.4 ± 5.44	17.3 ± 7.87	17.5 ± 8.72	<0.001***	0.765
Adipo-IR	45 ± 35.3	59.9 ± 35.8	71.7 ± 45.2	0.007**	43.7 ± 30.4	74.0 ± 70.7	71.7 ± 52.5	0.003**	0.806
HOMA-IR	3.17 ± 3.17	3.98 ± 2.24	5.05 ± 3.50	0.002**	2.95 ± 1.53	5.19 ± 3.11	4.76 ± 3.60	0.001**	0.476

**Abbreviations**: BMI, body mass index; AST, aspartate transaminase; ALT, alanine transaminase; GGT, gamma-glutamyl transferase; HDL, high-density lipoprotein; LDL, low-density lipoprotein; TG, triglycerides; FFA, free fatty acids; TB, total bilirubin; Alb, albumin; hs-CRP, high-sensitivity C-reactive protein; HA, hyaluronic acid; T3, triiodothyronine; HbA1c, glycated hemoglobin; FBG, fasting blood glucose; Adipo-IR, adipose tissue insulin resistance; HOMA-IR, homeostasis model assessment of insulin resistance. The mean and standard deviation are presented as the mean ± SD and the number of subjects and those percentages are expressed as n (%). Significant differences among subgroups were calculated using the Kruskal-Wallis test. *P < 0.05, **P < 0.01, ***P < 0.001.

### Associations between nutrient intake and NAFLD-associated clinical markers according to carbohydrate intake

**Figure 1.**
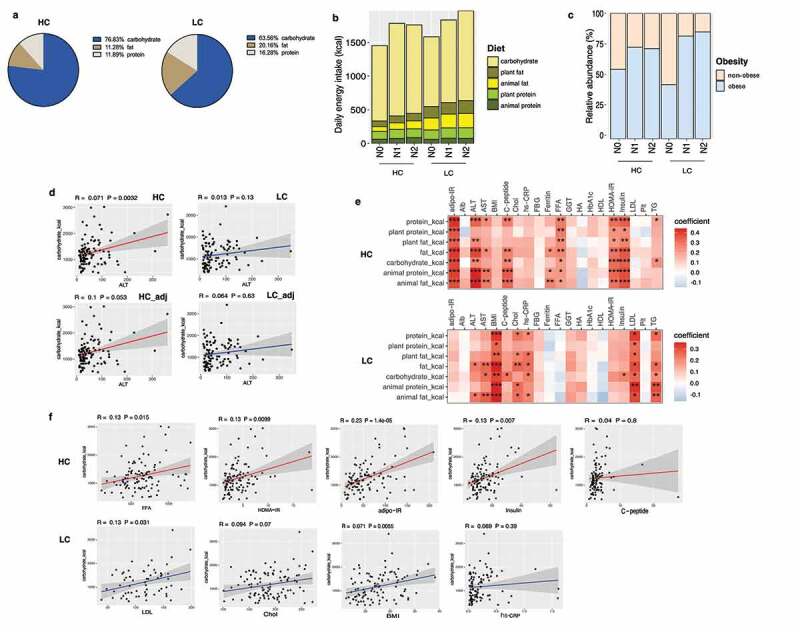
Stratification of the study population into high carbohydrate (HC) and low carbohydrate (LC) intake groups by carbohydrate intake and associations between nutrient intake and clinical markers in these groups. (a) Percentage of energy intake from carbohydrates, fats, and proteins in the HC and LC groups. (b) Absolute amounts of nutrient intake (in kcals) in each group, stratified by carbohydrate intake (%) and the NAFLD activity score (NAS). (c) The proportion of participants classified as obese population in each group (non-obese, BMI <25; obese, BMI ≥25 kg/m^2^), stratified by carbohydrate intake (%) and NAS. (d) Linear regression models (with 95% confidence interval bands highlighted in gray) between the levels of alanine transaminase (ALT) and carbohydrate intake (in kcals) with and without adjustment for BMI, age, and sex (HC, upper panel, red line; LC, lower panel, blue line). (e) Heatmap displaying the significant correlations between clinical markers and the intake of nutrients (*P < .05, **P < .01, ***P < .001). Positive correlations are expressed in red and negative correlations are in blue (HC, upper panel; LC, lower panel). (f) Linear regression models (with 95% confidence interval bands highlighted in gray) between clinical markers and the intake of carbohydrates (in kcals) after adjusting for BMI, age, and sex (HC, upper panel, red line; LC, lower panel, blue line).

**Abbreviations**: adipo-IR, adipose insulin resistance; Alb, albumin; ALT, alanine transaminase; AST, aspartate transaminase; BMI, body mass index; C-peptide, connecting peptide; Chol, cholesterol; hs-CRP, high-sensitivity C-reactive protein; FBG, fasting blood glucose; FFA, free fatty acids; GGT, gamma-glutamyl transferase; HA, hyaluronic acid; HbA1c, glycated hemoglobin; HDL, high-density lipoprotein; HOMA-IR, homeostasis model assessment for insulin resistance; LDL, low-density lipoprotein; Plt, platelet; TB, total bilirubin; TG, triglycerides.

On average, the HC group consumed 76.83% of daily total energy from carbohydrate, 11.28% from fat, and 11.89% from protein, while the LC group consumed 63.56% of daily total energy from carbohydrate, 20.16% from fat, and 16.28% from protein (Figure 1a). In terms of the absolute amount of each nutrient consumed, the LC group consumed higher amounts of fat, animal fat, plant fat, protein, animal protein, and plant protein than the HC group (Figure 1b, Supplementary Table 3).

BMI did not significantly differ between the HC and LC groups (Table 1); The percentage of obese NASH individuals was higher in the LC group (N0 [43%], N1 [81%], and N2 [85%]) than the HC group (N0 [55%], N1 [72%], and N2 [71%]) (Figure 1c). In the LC group, body fat mass and visceral, subcutaneous, and total abdominal adipose tissue areas significantly increased with worsening the histological severity of NAFLD (Supplementary Figure 1).

Carbohydrate consumption was positively correlated with serum alanine transaminase (ALT), a liver damage marker only in the HC group. After adjusting for potential confounding factors, the positive correlation between carbohydrate intake and ALT in the HC group remained marginally significant (*P* = .053) (Figure 1d). Significant positive correlations between the surrogate markers of insulin resistance (homeostasis model assessment of insulin resistance [HOMA-IR] and adipose tissue insulin resistance [adipo-IR]) with carbohydrate consumption were found only in the HC group. In contrast, obesity and lipid metabolism-associated clinical markers (low density lipoprotein [LDL]-cholesterol and TG) were significantly correlated with carbohydrate consumption in the LC group (Figure 1e). These results were confirmed by a linear regression model adjusted for BMI, age, and sex. Carbohydrate intake significantly predicted FFA concentrations, HOMA-IR, adipo-IR, and insulin levels in the HC group, whereas carbohydrate intake significantly predicted LDL-cholesterol levels and BMI in the LC group (Figure 1f).

### NAFLD activity-associated alterations in gut microbial diversity and composition in the HC group

**Figure 2.**
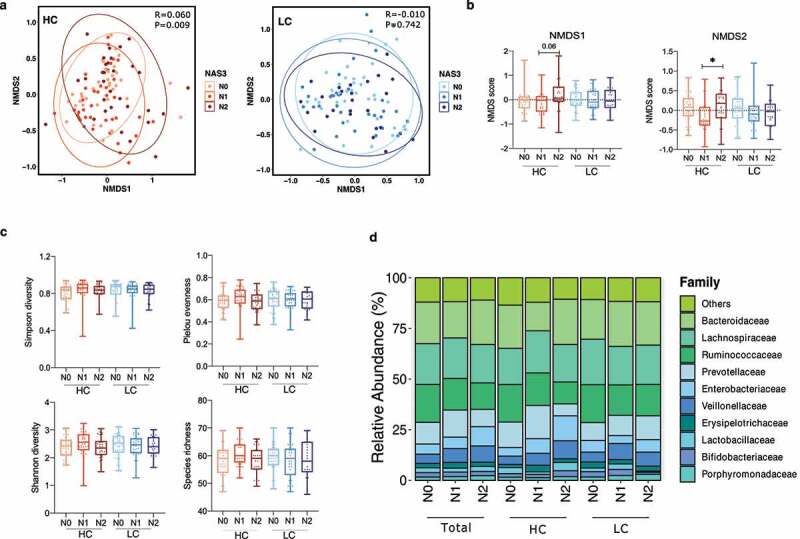
Alterations in the diversity and composition of the gut microbiome according to NAFLD severity in the high carbohydrate (HC) and low carbohydrate (LC) intake groups. (a) Non-metric multidimensional scaling (NMDS) plot based on the Bray-Curtis distance showing the beta diversity of the gut microbiome composition at the genus level (HC: N0, yellow; N1, Orange; N2, red and LC: N0, light sky blue; N1, dark blue; N2, navy). (b) NMDS plot from (A) visualized as Tukey’s box and whisker plot, showing the median and upper and lower quantiles. The nonparametric Kruskal-Wallis test and Dunn’s multiple comparisons test were used for statistical analysis. (c) Alpha diversity in each group (upper left, Simpson’s diversity index; upper right, Pielou’s evenness index; lower left, Shannon diversity index; lower right, species richness). (d) Relative abundances of the top 10 family taxa in the total, HC, and LC groups stratified by the NAFLD activity score.

In the HC group, the composition of gut microbiota varied significantly according to the NAS (*P* = .009), but this pattern was not exhibited in the LC group (*P* = .742) (Figure 2a). Subsequent analysis of nonmetric multidimensional scaling (NMDS) scores showed a significant difference between the N1 and N2 groups (*P* = .0195) (Figure 2b).

However, alpha diversity, measured using four indices (Simpson’s diversity index, Pielou’s evenness index, the Shannon diversity index, and species richness), did not significantly differ among the NAS subgroups in either the HC or LC group (Figure 2c). The top 10 microbial taxa enriched or depleted by NAFLD severity in all participants, the HC group, and the LC group were visualized using stacked bar plots (Figure 2d).

### NAFLD activity-associated alterations in the relative abundances of gut microbial taxa in the HC group

**Figure 3.**
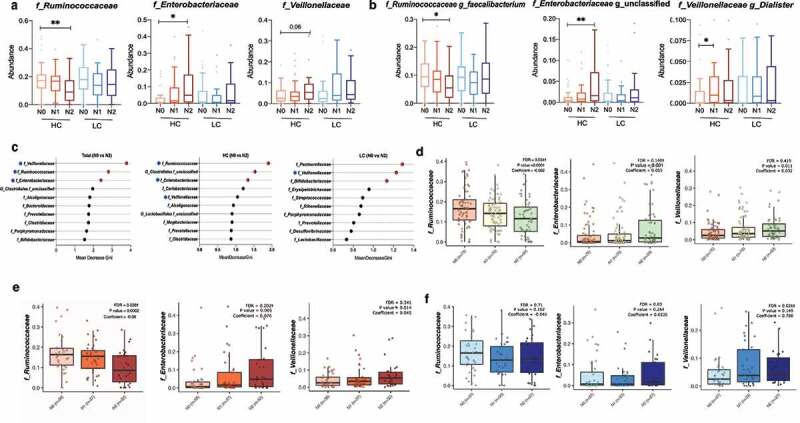
**Changes in the relative abundance of the gut microbial taxa according to NAFLD severity in the high carbohydrate (HC) and low carbohydrate (LC) intake groups**. (a) The abundance of three representative family taxa and (b) three representative genus taxa are depicted. Nonparametric Kruskal-Wallis tests and Dunn’s multiple comparisons tests were used in the statistical analysis. (c) Ranking of the family taxa according to the mean decrease in the Gini coefficient in the random forest model to predict the NAFLD activity score. (red circle, top 3 families defined by the random forest model; blue pentagon, three representative family taxa) (d–f) Regression analysis was used to determine the abundance of the *Ruminococcaceae, Enterobacteriaceae*, and *Veillonellaceae* families after adjusting for confounding factors including BMI, age, and sex. Multivariate association analysis was performed using MaAsLin2 with adjustment for multiple comparisons. (d) Total: *P* = .0001, q = 0.034; *P* = .001, q = 0.140; *P* = .011, q = 0.419. (e) HC: *P* = .002, q = 0.035; *P* = .005, q = 0.202; *P* = .014, q = 0.341. (f) LC: *P* = .102, q = 0.71; *P* = .264, q = 0.84; *P* = .149, q = 0.026. (*P < .05, **P < .01, ***P < .001, ^†^FDR<0.25).

Univariate analysis revealed a significant decrease in the abundance of *Ruminococcaceae* family (*P* = .006) and a significant increase in the abundance of *Enterobacteriaceae* family (*P* = .010) with increasing NAFLD activity (N2 vs. N0) in the HC group, and a marginally significant increase in the abundance of *Veillonellaceae* in individuals with definite NASH (N2) compared to non-NASH individuals (N0) (*P* = .06). No significant alterations in the abundance of microbial taxa were found in the LC group (Figure 3a). These trends were maintained at the genus level in the HC group (Figure 3b): compared with subjects without NASH, NASH subjects had a significant decrease in the abundance of *Faecalibacterium* (N2 vs. N0), and a significant increase in the abundance of unclassified *Enterobacteriaceae* (N2 vs. N0) and *Dialister* (N1 vs. N0).

The predictive validity of microbial taxa for NAFLD was tested using a random forest model (Figure 3c). The most critical microbial variables in the total population were the *Veillonellaceae, Ruminococcaceae*, and *Enterobacteriaceae* families. The *Ruminococcaceae* and *Enterobacteriaceae* families were also crucial microbial variables in the HC group. Microbial variables selected for the prediction of NAFLD in the LC group were not significantly altered by NAFLD activity. Multivariate analysis was performed using MaAsLin2^19^ after adjustment for BMI, age, and sex. Similar to the unadjusted data, in the HC group, the abundance of *Ruminococcaceae* was significantly depleted with increasing NAFLD activity, while the abundance of *Enterobacteriaceae* was significantly enriched (Figure 3d-f). Similar significant associations were not found in the LC group.

To analyze the functional changes in the gut microbiome in the HC and LC groups according to NAFLD severity, the functional prediction analysis was conducted. As a result of linear discriminant analysis effect size (LEfSe), a total of 287 Kyoto Encyclopedia of Genes and Genomes (KEGG) categories and 16 KEGG pathways were identified as significantly differing between the non-NASH (N0) and NASH (N1 and N2) subgroups within the HC group. On the other hand, no differences in the functional changes in KEGG categories between the non-NASH and NASH subgroups were observed, and 6 KEGG pathways were identified as significant in the LC group. The KEGG pathways found to be significant in the HC group are visualized in supplementary figure 2. Notably, the majority of KEGG pathways that were enriched in the NASH subgroup within the HC group were related to microbial carbohydrate metabolism, such as ‘phosphotransferase system PTS’, ‘glycerolipid metabolism’ and ‘fructose and mannose metabolism’.

### Effect of carbohydrate intake on microbial diversity and composition in the non-NAFLD population

**Figure 4.**
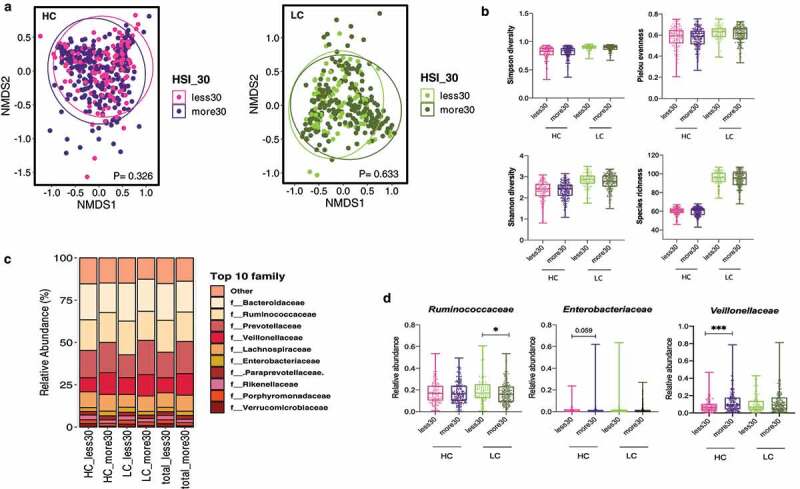
**Alterations in the diversity and composition of the gut microbiome according to nonalcoholic fatty liver disease (NAFLD) risk in high carbohydrate (HC) and low carbohydrate (LC) intake groups from the Health Twin cohort**. A hepatic steatosis index (HSI) of 30 IU/L was used as the cutoff for determining NAFLD risk in the HC and LC groups (HC, n = 382; LC, n = 300). (a) NMDS plot showing the beta diversity of the gut microbiome composition in subjects with and without liver injury. NMDS scores were calculated using Bray-Curtis distance (HC group: HSI <30, pink; HSI ≥30, purple; LC group: HSI <30, yellow-green; HSI ≥30, dark green). (b) Alpha diversity was calculated using four indices (upper left, Simpson’s diversity index; upper right, the Shannon diversity; lower left, Pielou’s evenness index; lower right, species richness). (c) Relative abundances of the top 10 families are visualized in stacked bar plots for all patients as well as the HC and LC groups, stratified by liver injury severity. (d) Univariate analysis of three key microbial families using nonparametric Mann-Whitney tests and Dunn’s multiple comparisons tests (*P < .05, **P < .01, ***P < .001).

To investigate the effect of an HC diet on the gut microbiome in the non-NAFLD population, gut microbiome data from the Korean healthy twin cohort were analyzed (n = 682) (Figure 4). Study subjects were divided into the HC and LC groups using a cutoff of 70% energy intake from carbohydrates (HC group, n = 382; LC group, n = 300) (Supplementary Figure 3A). Furthermore, both groups were subdivided into two subgroups according to the hepatic steatosis index (HSI) (HC group: HSI ≥30, n = 246; HSI <30, n = 135 and LC group: HSI ≥30, n = 211; HSI <30, n = 88). The population characteristics of the HC and LC groups in the healthy twin cohort are described in supplementary information (Supplementary Table 5, Supplementary Figure 3).

The composition and alpha diversity of the gut microbiome did not significantly differ by the HSI in the healthy twin cohort, regardless of carbohydrate intake (Figures 4 A and b). Among the three crucial microbial families (*Ruminococcaceae, Enterobacteriaceae, and Veillonellaceae*), the enrichment of the abundance of *Veillonellaceae* in individuals with high HSI was evident in all participants as well as in the HC and LC groups (Figure 4c). This observation was also confirmed in the HC group by univariate analysis (Figure 4d).

### Differential regulation of DNL-related liver transcripts in the HC and LC groups

NAFLD-associated single nucleotide polymorphisms (SNPs) were analyzed to understand the genetic background of subjects (Supplementary Table 6). Of the five major SNPs (*PNPLA3*_rs738409 C > G, *TM6SF2*_rs58542926 C > T, *SREBF2*_rs133291 C > T, *MBOAT7*_rs641738 C > T, and *HSD17B13*_rs72613567 adenine insertion [A-INS]), only the prevalence of *PNPLA3*_rs738409 C > G significantly differed among the NAS subgroups in both the HC and LC groups. There were no significant differences in the minor allele frequency of *PNPLA3*_rs738409 between the HC and LC groups (*P* = .612), suggesting that the differences between the HC and LC groups were not attributable to genetic factors.

**Figure 5.**
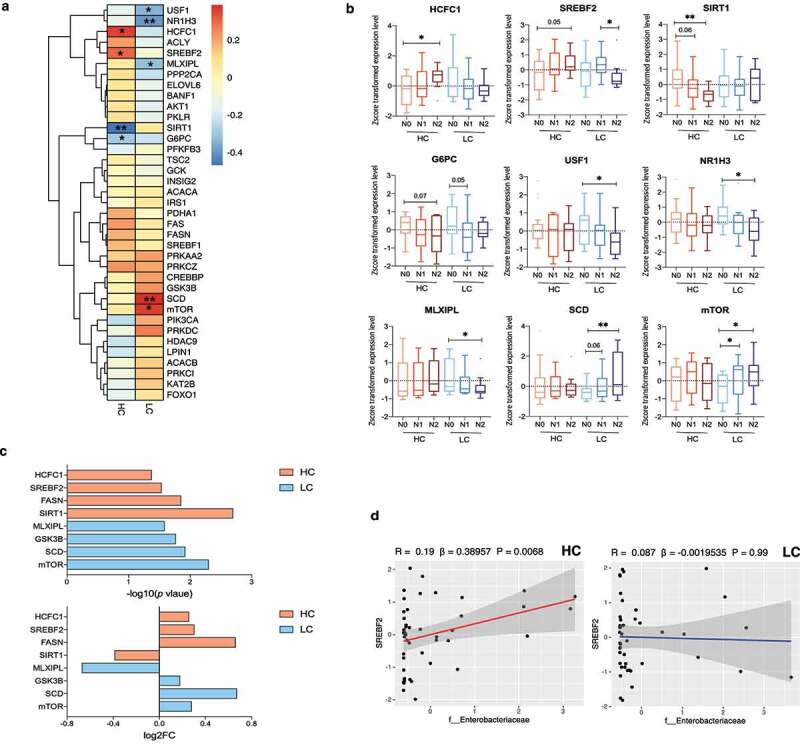
Changes in *de novo* lipogenesis (DNL)-related transcripts in the high carbohydrate (HC) intake group. (a) Heatmap displaying the correlation between DNL-related genes and the NAFLD activity score (NAS) in the HC and LC groups. Statistical analysis was performed using transformed z scores and Spearman’s rank correlation analysis. (b) Z score-transformed expression levels of predefined transcripts were compared between the HC and LC groups according to NAFLD severity using an one-way analyses of variance and the FDR correction for multiple comparisons. (c) Differentially expressed genes were defined using the R package DEseq2. The upper panel indicates log_10_ transformed *P* values and the lower panel indicates log_2_ transformed fold changes. (d) The linear regression model depicts the relationship between *SREBF2* and *Enterobacteriaceae* after adjustment for BMI, age, and sex (*P < .05, **P < .01, ***P < .001).

Next, we analyzed liver transcripts related to hepatic DNL in the biopsy-proven NAFLD cohort (n = 90) (Figure 5). We investigated whether an HC diet was associated with upregulation of DNL due to increased glucose availability in the liver. A total of 37 DEseq2-normalized transcripts were selected and compared between the HC and LC groups according to NAFLD activity (Supplementary Table 7). We performed Spearman’s rank correlation analysis to analyze the relationship between the selected transcripts and the NAS in the HC and LC groups. In the HC group, *G6PC* and *SIRT1* were negatively correlated with the NAS (*P* = .049 and *P* = .001), while *HCFC1* and *SREBF2* were positively correlated with the NAS (*P* = .016 and *P* = .041). In the LC group, *USF1, NR1H3*, and *MLXIPL* were inversely correlated with the NAS (*P* = .021, *P* = .008, and *P* = .037), whereas *SCD* and *mTOR* were positively correlated with the NAS (*P* = .009 and *P* = .017) (Figure 5a).

Significant correlations were confirmed by comparing z score-transformed expression levels of the corresponding genes (Figure 5b). In the HC group, *HCFC1* expression was higher and *SIRT1* expression was lower in N2 subjects than in N0 subjects (*P* = .018 and *P* = .001, respectively). Although differences in the expression of *SREBF2* and *G6PC* did not reach statistical significance, higher *SREBF2* expression and lower *G6PC* expression were observed with increasing NAFLD activity (N2 vs. N0; *P* = .057 and *P* = .072, respectively). The expression of *USF1, NR1H3*, and *MLXIPL* was lower (*P* = .022, *P* = .009, and *P* = .045, respectively) and the expression of *SCD* and *mTOR* was higher with increasing NAFLD activity in the LC group (*SCD, P* = .009 [N2 vs. N0]; *mTOR, P* = .035 [N0 vs. N1]; and *P* = .022 [N0 vs. N2]). Among the corresponding transcripts in the HC group, *SREBF2, SIRT1* and *HCFC1* significantly differed between the HC and LC groups in the definite NASH subgroup (N2) (Supplementary Figure 4).

We identified differentially expressed genes between the N0 vs. N1+ N2 subgroups (Figure 5c). Specifically, *SIRT1, FASN, SREBF2*, and *HCFC1* were differentially expressed in the HC group (*P* = .002, *P = *.014, *P* = .029, and *P* = .042, respectively) and the expression of *mTOR, SCD, GSK3B*, and *MLXIPL* were differentially expressed in the LC group (*P* = .005, *P* = .012, *P* = .017, and *P* = .026, respectively). Among the analyzed transcripts, we focused on the association between *SREBF2* and the abundance of *Enterobacteriaceae* (Figure 5d, Supplementary Figure 5). A linear regression model adjusted for BMI, age, and sex revealed that the expression of *SREBF2* was predictive of *Enterobacteriaceae* abundance in the HC group (*P* = .006). This suggests a close relationship between NAFLD activity, DNL, and the gut microbiome community under HC diet conditions.

### Prediction of NASH using three gut microbial families in the HC group

**Figure 6.**
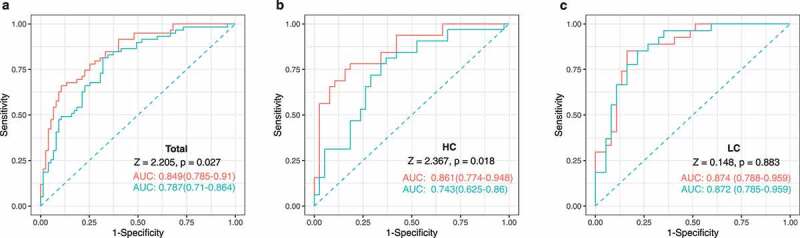
**Noninvasive multidimensional prediction of nonalcoholic steatohepatitis (NASH) using three microbial families and potential clinical variables in all subjects as well as the high carbohydrate (HC), and low carbohydrate (LC) intake groups (non-NASH vs. NASH)**. The receiver-operating characteristic (ROC) curve was designed for detecting NASH among study subjects with biopsy-proven NAFLD. Area under receiver-operating characteristic curves (AUROC) analyses including clinical variables (BMI, age, and sex) (blue) vs. those also including the three microbial families (*Enterobacteriaceae, Ruminococcaceae*, and *Veillonellaceae*) in addition to clinical variables (BMI, age, and sex) (red) plotted for the diagnosis of NASH in (a)all subjects as well as, (b) the HC, and (c) LC groups. *P* values were calculated using the DeLong test.

We performed the area under the receiver-operating characteristic curve (AUROC) analysis to evaluate whether specific gut microbial families predict the presence of NASH among subjects with biopsy-proven NAFLD. Three microbial families, *Enterobacteriaceae, Ruminococcaceae*, and *Veillonellaceae*, which were present in differing amounts according to NAFLD activity in the HC group, were incorporated into the prediction model (Figure 6). For NASH diagnosis, the addition of these microbial families to the prediction model including BMI, age, and sex yielded an AUC of 0.861 in the HC group (95% CI, 0.774–0.940), which was significantly higher than that of the prediction model including only BMI, age, and sex (AUC = 0.743; 95% CI, 0.625–0.860; *P* = .018 by DeLong test) (Figure 6b). However, the addition of those microbial families did not significantly improve the predictive validity of the model for detecting NASH in the LC group (AUC = 0.874 [microbes-BMI-age-sex]; AUC = 0.872 [BMI-age-sex]; 95% CI, 0.735–0.937; *P* = .883 by DeLong test) (Figure 6c).

### Causal effects of clinical markers, gut microbes, and gene expression on NAFLD pathogenesis

**Figure 7.**
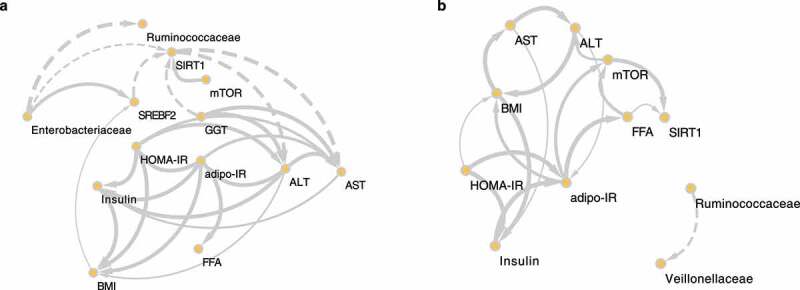
**The causal relationships among nonalcoholic steatohepatitis (NASH)-related microbes, transcripts, and clinical factors in the high carbohydrate (HC) and low carbohydrate (LC) intake groups**. Causal inference of inverse, normally transformed microbiome families (*Ruminococcaceae* and *Enterobacteriaceae*), transcripts (*SREBF2, SIRT1*, and *mTOR*), and clinical variables (ALT, AST, adipo-IR, HOMA-IR, insulin, FFA, and BMI) were calculated using kernel causality analysis; significant causal correlations were visualized using Cytoscape. Causality of variables in the (a) HC and (b) LC groups. Solid line indicates a positive correlation and the dashed lines indicates a negative correlation. The thickness of the line indicates the degree of statistical significance (thin: less significant *P* values; thick: more-significant *P* values).

**Abbreviations**: *SIRT1*, silent mating type information regulation 2 homolog 1; *mTOR*, mechanistic target of rapamycin kinase; *SREBF2*, sterol regulatory element-binding transcription factor 2; GGT, gamma-glutamyl transferase; HOMA-IR, homeostasis model assessment of insulin resistance; adipo-IR, adipose tissue insulin resistance; BMI, body mass index; FFA, free fatty acids; ALT, alanine transaminase; AST, aspartate transaminase.

To determine causality among several variables, we used kernel causality analysis, which identifies plausible causal pathways between two variables using generalized correlation coefficients^20^(Figure 7). The descriptive source data are shown in Supplementary Table 8. Differences in the causality of variables between the HC and LC groups were observed. In the HC group, we identified positive causation between insulin resistance-related markers, such as HOMA-IR and adipo-IR, and liver damage markers, such as ALT, aspartate transaminase (AST), and gamma-glutamyl transferase (GGT). Additionally, there was positive causation between the abundance of *Enterobacteriaceae* and a DNL regulator, *SREBF2*; both *Enterobacteriaceae* and *SREBF2* were involved in reducing *SIRT1*, a lipid biosynthesis suppressor gene.

## Discussion

The current study demonstrated that in individuals with NAFLD who consume an HC diet (but not a LC diet), alterations in the composition of the gut microbiome are associated with NAFLD activity. In particular, the abundances of three key microbial families (*Veillonellaceae, Ruminococcaceae*, and *Enterobacteriaceae*) differed among the NAS subgroups within the HC group, such that a set of these microbes served as an accurate tool for diagnosing NASH in the HC group. However, HC consumption did not impact the gut microbiome composition in the absence of NAFLD, according to an independent non-NAFLD cohort. In the transcriptomic analysis of human liver tissues from the subjects with biopsy-proven NAFLD, we found four differentially expressed genes (*G6PC, SIRT1, HCFC1*, and *SREBF2*) between non-NAFLD controls and NASH subjects who consumed HC. We then explored the direction of causality of the three microbial families, eight clinical markers, and three transcriptomes using kernel causality analysis.

HC consumption was positively correlated with an insulin resistance phenotype in subjects with biopsy-proven NAFLD. Given the very high proportion of energy intake from carbohydrates in the HC group, it is plausible that chronic high consumption of carbohydrates worsens insulin resistance.^21^ According to a Japanese population-based study, high consumption of white rice, a primary carbohydrate source in East Asia, was positively associated with the risk of type 2 DM.^22^In addition, a high HC-intake-to-energy ratio was positively associated with a high prevalence of NAFLD.^23^ Mechanistically, several studies have reported a close relationship between hepatic steatosis and hepatic insulin resistance.^24–26^^,^ Although a causal relationship among HC consumption, NAFLD, and DM remains to be elucidated, the results from our kernel causality analysis offer some clues to the relationship among these conditions.

In the current study, differences in three NAFLD-associated microbial taxa were identified in the HC group by multivariate analysis. In a recent study, we reported that the *Veillonellaceae* and *Ruminococcaceae* families contribute to significant fibrosis in nonobese subjects with NAFLD,^11^ while the *Enterobacteriaceae* family was not significantly altered by fibrosis severity after adjustment for DM.^11^ This relationship identified among the *Enterobacteriaceae* family, DM, and an HC diet was supported by the current study findings that the surrogate markers of diabetes were associated with HC consumption in NAFLD subjects. Indeed, several studies have found that the abundance of *Enterobacteriaceae* is enriched in adipose tissue and plasma samples from individuals with DM^27^ and in feces from NAFLD subjects.^28,29^ Our results led to the conclusion that *Enterobacteriaceae* forms a close multifactorial association with DM and NAFLD. In addition, the functional analysis indicated that the microbial carbohydrate uptake system (specifically, the phosphotransferase system) was enriched in the NAFLD subgroup within the HC group. This result was consistent with a recent study that two strains belonging to the family *Enterobacteriaceae* strongly compete for carbohydrate, even leading to colonization resistance.^30^ Thus, the enrichment of the abundance of *Enterobacteriaceae* with worsening NAFLD severity observed in the HC group might be attributed to ample opportunities for carbohydrate utilization.

An association between the *Ruminococcaceae* family and NASH has been identified in several human studies.^29,31^ Children with NASH showed a significant reduction in the abundance of *Ruminococcaceae* compared with healthy children;^29^ additionally, a UK study also found that the abundance of *Ruminococcaceae* decreased in patients with biopsy-proven NASH.^31^ In the current study, the genus *faecalibacterium* in the family *Ruminococcaceae* may largely account for the decreased abundance of *Ruminococcaceae* in patients with advanced NAFLD in the HC group. Indeed, hepatic DNL is significantly decreased by the short-chain fatty acids produced by *faecalibacterium*^32^ and the abundance of *Faecalibacterium prausnitzii* has been linked to liver fat accumulation as measured by magnetic resonance spectroscopy.^33^ These results highlight the dynamic crosstalk between *Ruminococcaceae*, DNL, and NAFLD severity.

Alterations in the gut microbiome according to NAFLD severity in the HC group were confirmed by comparing the gut microbiome composition of the NAFLD cohort with that of the non-NAFLD cohort. The gut microbiome composition of the non-NAFLD cohort, unlike that of the NAFLD cohort, stratified by the HSI in the HC group was not significantly altered by the HSI. This indicates that the contributing factor to the alteration of the gut microbiome composition is the interaction between NAFLD severity and an HC diet rather than their individual effects. On the other hand, univariate and multivariate analyses of the gut microbiome in the NAFLD cohort showed that the abundance of *Veillonellaceae* was not significantly enriched with worsening NAFLD severity in the HC group. However, the abundance of *Veillonellaceae* significantly increased with high HSI (>30) in the HC group of the non-NAFLD twin cohort. Thus, we speculate that the enrichment of the abundance of *Veillonellaceae* in the HC group is related with hepatic steatosis but not with severe NASH. Although several previous studies reported the association between *Veillonellaceae* and NAFLD,^11,34^ further studies on the colonization of *Veillonellaceae* during the certain stages of NAFLD under HC consumption are warranted.

To investigate whether HC consumption alters hepatic DNL-related gene expression, we performed liver transcriptomic analysis in 90 subjects with biopsy-proven NAFLD. Among the 37 genes that encode proteins involved in DNL, we identified three central DNL regulators (*SREBF2, SIRT1*, and *mTOR*) that are significantly associated with NASH pathogenesis as well as carbohydrate metabolism. Compared with non-NASH patients (the N0 subgroup), definite NASH patients (N2) in the HC group showed higher expression of *SREBF2* and lower expression of *SIRT1*; overall, NASH patients (N1 and N2 subgroups) in the LC group showed higher expression of *mTOR*.

The expression of *SREBF2*, the gene encoding sterol regulatory element-binding protein 2 (SREBP2), was upregulated upon increasing NAFLD severity in the HC group. *SREBPs* can activate the transcription of genes involved in the synthesis of cholesterol, fatty acids, and phospholipids.^35,36^ The association between *SREBF2* and NASH is well established: *SREBF2* mRNA levels are three times higher in NASH patients than in healthy controls, and acyl-CoA cholesterol acyltransferase was 1.5-fold increased.^37^ Thus, chronic carbohydrate consumption may stimulate *SREBP* expression and the subsequent upregulation of hepatic DNL, leading to excessive lipid accumulation and the onset of NAFLD. We also demonstrated that the diet-induced increase in hepatic *SREBF2* expression was positively associated with the abundance of *Enterobacteriaceae*. Although a direct interaction between *SREBP2* and *Enterobacteriaceae* has not yet been established, our results provide novel insights into the gut microbe mediation of DNL, which occurs via regulation of hepatic gene expression in relation to an HC diet.

The expression of *SIRT1*, a potential inhibitor of hepatic DNL, decreased with increasing NASH severity in the HC group. The function of *SIRT1* in preventing liver fat accumulation is well established in both mice and humans.^38,39^ A loss-of-function model using *SIRT1*-knockout mice displayed hepatic insulin resistance as well as increased hepatic lipogenesis;^38^ hepatocytes differentiated from human induced pluripotent stem cells with deletion of *SIRT1* exhibited a NASH phenotype, including steatosis and inflammation.^39^ Due to its responsiveness to hepatic insulin, *SIRT1* is regarded as a potential therapeutic target for treating type 2 DM.^40^ Thus, reduced expression of *SIRT1* may be derived from impaired hepatic lipid and glucose homeostasis.

In the current study, *mTOR*, an insulin-signaling regulator^41^and a key player in adipogenesis,^42^ was expressed at higher levels in patients with advanced NAFLD in the LC group. The LC group consumed a relatively high-fat diet and contained a higher proportion of obese people than the HC group. An *in vivo* study using adipose tissue-specific *mTORC1*-knockout mice revealed that loss of *mTORC1* induces resistance to high-fat diet-induced obesity.^43^ Moreover, *mTORC1* is highly activated in the liver of diet-induced obese animals with impaired insulin signaling.^44,45^ Activation of *mTOR* is associated with the development of metabolic syndrome and NASH in humans and animals.^46^Thus, there seems to be an intricate relationship among *mTOR*, obesity, and NASH, suggesting that the increased *mTOR* expression observed in individuals with NASH in the LC group might be attributed to the high prevalence of obesity in this group.

Finally, we integrated clinical, microbial, and transcriptomic data to identify causality among variables involved in the etiology of NAFLD associated with HC and LC diets. As expected, the surrogate markers of insulin resistance displayed positive causal effects on NASH biomarkers. Moreover, GGT was the causal agent that reduced *SIRT1* expression in the HC group. GGT is inversely associated with insulin sensitivity,^47^ and insulin resistance induces hepatic DNL in NAFLD.^9^ In addition, *Enterobacteriaceae* play a central role in regulating DNL-associated genes by stimulating *SREBF2* and inhibiting *SIRT1* expression. A previous study using germ-free and specific pathogen-free mice demonstrated that intestinal microbes promote hepatic fatty acid metabolism via the transcription of DNL-associated genes, including *SCD1* and *ELOVL5*, which are modulated by SREBP1C.^48^ Moreover, knockdown removal of *SIRT1* in mice alters the gut microbiota, leading to intestinal inflammation,^49^ which may partly explain the crosstalk between the hepatic transcriptome and the intestinal microbiota. Thus, impaired insulin sensitivity due to extreme HC consumption and lipid accumulation-associated liver damage may result from the inactivation of DNL suppressors through microbial reshaping.

The current study utilized the nested case-control data from a well-characterized biopsy-proven NAFLD cohort, providing the unique insight that the progression of NAFLD in the HC group is a modulating factor for the alteration of the gut microbiome and that these changes in the gut microbiome contribute to host hepatic metabolism in an Asian population. Nevertheless, this study has several limitations. First, the cross-sectional design of our study may preclude us from drawing a number of causal mechanistic insights between nutrition and NAFLD. However, we adopted the robust statistical methods to address this issue. We compared the gut microbiome data obtained from 16S rRNA amplicon sequencing between subjects with HC and LC diets and also scrutinized hepatic transcriptomic data to identify underlying mechanisms. Moreover, we attempted to verify the causality of defined factors using kernel causality analysis. Although kernel causality analysis is a useful method to gain an insight into nonexperimental causality and has been used for a long time in various fields including the field of microbiology,^50^further interventional studies, including animal experiments and human clinical trials, are warranted to validate our findings on the macronutrient-centered gut-liver axis. Second, the pathogenesis of NAFLD in the LC group remains unclear. Due to the typical dietary pattern of an Asian NAFLD population,^51,52^ even the LC group in the current study consumed a relatively high level of carbohydrates (63.7%) compared with Western NAFLD populations (~44–46%).^53,54^ Thus, it was difficult to clearly distinguish between the effects of HC and LC diets in the current study; nevertheless, we identified obesity as the main contributing factor to NAFLD severity in the LC group.

In summary, based on a multidisciplinary approach, we highlight that habitual HC consumption may be associated with adverse hepatic metabolism and NAFLD severity, which result from alterations in the gut microbiome. In particular, we demonstrated that HC intake is significantly associated with insulin resistance markers and may lead to a prominent shift in microbial diversity and the abundance of specific taxa according to NAFLD activity. We also confirmed that the addition of microbial taxa may significantly improve the prediction of NASH in the HC group. Therefore, the enrichment of pathogenic intestinal microbiota and the depletion of protective gut microbiota by increased NAFLD severity in the HC group seem to be associated with host hepatic metabolism through the transcriptional activation of hepatic DNL.

## Methods

### Study population and liver histology

This cross-sectional study was performed using the ongoing Boramae NAFLD cohort (NCT02206841).^11,55,56^ This study was performed in accordance with the ethical guidelines of the 1975 Declaration of Helsinki for the participation of human subjects. This study protocol was approved by the Institutional Review Board of Boramae Medical Center (IRB No. 26–2017-48). All study subjects provided written informed consent. The liver specimens were obtained using 16 G disposable needles, then fixed in 4% formalin and embedded in paraffin. The specimens with 20 mm in length and 3 mm in thickness were stained with hematoxylin and eosin and Masson’s trichrome. Liver histology was assessed using the NAFLD activity scoring system.^57^ The combination of scored steatosis, lobular inflammation, and ballooning was considered for categorizing study subjects. Patients with an NAS of 0–2 were classified as non-NASH (N0), those with an NAS of 3–4 were classified as having borderline NASH (N1), and those with an NAS of 5–8 were classified as having definite NASH (N2).^58^ Participants with type 1 DM and advanced fibrosis (≥F3) were excluded from this study. Clinical, biochemical, and genetic parameters were evaluated as previously described elsewhere.^11,55,56^

### Dietary analysis

Usual dietary intake was assessed using 103 items from a food frequency questionnaire (FFQ).^59^ The frequency of servings during the last year was classified into nine categories: never, once a month, 2–3 times a month, 1–2 times a week, 3–4 times a week, 5–6 times a week, once a day, twice a day, and three times a day. Portion size was categorized as small, medium, or large. Each individual’s daily nutrient intake was calculated using CAN-Pro 5.0 (The Korean Nutrition Society, Seoul, Korea). The calculated daily absolute amount of carbohydrate intake (g) was converted to energy consumed from carbohydrate by multiplying by four calories. Carbohydrate consumption as a proportion of daily energy intake was determined by dividing calories from carbohydrate by the total energy intake.

### Biomarker measurements

The HOMA-IR and adipo-IR were measured as described in previous studies.^60,61^ Liver biopsy was only indicated for study subjects who had at least two risk factors. The risk factors included high TG levels, low high-density lipoprotein cholesterol levels, abdominal obesity, hypertension, DM or insulin resistance, and clinically suspected NASH or hepatic fibrosis.^55,56^

### Microbiome data extraction from external cohorts

Curated metagenomic data from the NAFLD cohort were utilized in this study.^11^ Raw data sequencing was processed using the QIIME pipeline (v 1.8.0).^62^ Selection and assignment of operational taxonomic unit (OTU) were performed using the gg_13_5 Greengenes database at 97% similarity level.^63^ Representative sequences were selected and aligned using the UCLUST software and the PyNAST algorithm.^64^ OTUs were assigned to taxa using the ribosomal database project classifier.^65, 66^ Chimeric sequences were removed using the ChimeraSlayer algorithm.^66^ The relative abundance tables at the family and genus levels were used for the microbiome analysis. All of the relative abundance tables were filtered at the 0.001% abundance level and 50% persistence level.

### Non-NAFLD cohort analysis

A total of 682 subjects were recruited from the Healthy Twin study, which was part of the Korean Genome Epidemiology study.^67^ The HC and LC groups in this current study were created in concordance with the main study. Control and NAFLD risk groups were determined according to the HSI.^68^ Detailed information on the sample collection, 16S rRNA sequencing targeting the V4 region, and bioinformatics processing is described elsewhere.^69^ In the current study, processed operational taxonomic unit tables at the family and genus levels were utilized for comparing the compositions of the gut microbiomes.

### Host genotyping

Single nucleotide polymorphisms (SNP) genotyping of the entire study population was performed using TaqMan 50 nuclease assays (Life Technologies, Carlsbad, CA) or Sanger sequencing (Macrogen, Inc, Seoul, South Korea) according to the manufacturer’s protocol. Hardy-Weinberg equilibrium was analyzed using the chi-square test. The following SNPs were selected and have been previously described:^55,70^ *PNPLA3*_rs738409 C > G,^71^ *TM6SF2*_rs58542926 C > T,^72^*SREBF2*_rs133291 C > T,^70^ *MBOAT7*_rs641738 C > T,^73^ and *HSD17B13*_rs72613567 adenine insertion (A-INS).^74^

### Liver transcriptome analysis

Total hepatic RNA was extracted using TRIzol reagent (Invitrogen, Carlsbad, CA), according to the manufacturer’s protocol. RNA quality was determined using the BioAnalyzer (Agilent Technologies, Inc., Santa Clara, CA). The mean RNA integrity number (RIN) was 8.35 (4.5–9.5). cDNA library construction was performed using a TruSeq Stranded Total RNA Sample Prep Kit (Illumina, Inc., San Diego, CA). Libraries were sequenced using the Illumina platform. Raw data in fastq format were processed using STAR^75^ and HTseq.^76^ After performing a quality control of the raw counts, normalization of raw count data was undertaken using DESeq2.^77^

### Bioinformatic and statistical analyses

Statistical comparisons between the HC and LC groups were conducted with the Kruskal-Wallis test and Dunn’s multiple comparisons test using GraphPad Prism software ver 8.0d (GraphPad Software, San Diego, CA). Four indices of alpha diversity were calculated with OTU tables at the genus level using the Vegan package in R.^78^ This package was also used to display NMDS plots of beta diversity. The distance between genera was calculated using the Bray-Curtis distance method, and differences between subgroups were evaluated using the analysis of similarities (ANOSIM) function. Random forest analysis was conducted using the RandomForestUtils package in R^79^ to determine which microbial families were predictive of NASH. To exclude the potential confounding factors (age, BMI, and sex) for identifying specific taxa associated with NASH at the family level associated with NASH in the HC and LC groups, multivariate association analysis was performed using the MaAsLin2 package in R.^19^ The functional pathway analysis was conducted using PICRUSt2, and the KEGG database was used to infer the metagenomes.^80^ LEfSe with a threshold LDA score of 2.0 was performed to identify the pathways that significantly differed between the non-NASH (N0) and NASH (N1 and N2) subgroups in the HC and LC groups, respectively (Galaxy platform, https://huttenhower.sph.harvard.edu/galaxy)^81^. To estimate the predictive power of the three microbial families (*Ruminococcaceae, Veillonellaceae*, and *Enterobacteriaceae*), AUROC analysis was performed using the pROC and multipleROC package in R.^82^ For causal inference between variables, kernel causality analysis was used. The principal concept of the kernel causality method is to invest kernel regression in both directions of two variables; the variable with the larger correlation coefficient is considered the ‘kernel cause’.^83^ This kernel cause was measured using the generalCorr package in R^84^ and visualized using Cytoscape (v3.8.0).^85^

## Supplementary Material

Supplemental Material

## Data Availability

The V4 16S rDNA metagenome sequence datasets obtained in this study have been deposited in the European Nucleotide Archive databases under the accession number ERP109777 (https://www.ebi.ac.uk/ena/browser/view/PRJEB27662). The sequences from the Twin cohort study used in this study were deposited in the European Nucleotide Archive under the study accession number ERP010289. (https://www.ebi.ac.uk/ena/browser/view/PRJEB9205). All other metadata that support the findings of this study are available from the corresponding author upon reasonable request.
