# Supplementary material for: Interaction effect between NAFLD severity and high carbohydrate diet on gut microbiome alteration and hepatic de novo lipogenesis
Source: Gut Microbes. 2022 May 29;14(1):2078612. doi: 10.1080/19490976.2022.2078612 (PMC9154801; doi:10.1080/19490976.2022.2078612)
Supplement: Supplemental Material [file KGMI_A_2078612_SM4505.zip › Suppl_data_KGMI_20220049_gut microbes_revision.docx]

**Supplementary information**

**Interaction effect between NAFLD severity and high carbohydrate diet on gut microbiome alteration and hepatic *de novo* lipogenesis**

**Methods**

**Transcripts selection**

Among the entire transcripts, 37 ones related to hepatic *de novo* lipogenesis (DNL) were selected. Normalized raw count data were z-score-transformed before performing statistical analysis. The comparison of each transcript among the subgroups of nonalcoholic fatty liver disease (NAFLD) severity in the high carbohydrate (HC) and low carbohydrate (LC) consumers was made using one-way ANOVAs and FDR multiple comparisons. The correlation analysis between transcripts and the NAFLD activity score (NAS) was performed using Spearman’s correlation in GraphPad Prism software Ver. 8.4.2 (GraphPad Software, San Diego, CA) and visualized using the pheatmap^1^ package in R (V 3.6.3)^2^. Differences in gene expression between non-NASH and NASH subgroups were analyzed using the DESeq2^3^ package in R^2^. Adjusted linear regression was plotted using the ggplot2^4^ package in R^2^.

A total of 37 target liver transcriptomes were selected based on the previous researches focused on carbohydrate-associated hepatic DNL. The 3 transcriptomes were selected for the glycolysis pathway: *GCK*^5^, *G6PC*^6^, and *PFKB3*^7^. *PDHA1*^8^ was selected for representing the TCA cycle-associated transcriptome. The 11 transcriptomes were chosen for explaining the hepatic DNL process: *ACLY*^9^, *ACACA*^9^, *ACACB*^10^, *CREBBP*^11^, *USF1*^12^, *HDAC9*^13^, *KAT2B*^14^, *FASN*^9^, *FAS*^13^, *SCD*^15^, and *ELOVL6*^16^. The negative regulators of DNL were *PRKAA2*^17^ and *SIRT1*^18^. The SREBP1c pathway induced by insulin was represented by the 15 transcriptomes: *SREBF1*^19^, *SREBF2*^20^, *PIK3CA*^21^, *AKT1*^22^, *PRKDC*^23^, *PRKCI*^23^, *mTOR*^22^, *LPIN1*^23^, *IRS1*^23^, *GSK3B*^23^, *TSC2*^23^, *INSIG2*^23^, *FOXO1*^24^, and *NR1H3*^25^. The 5 transcriptomes were chosen for explaining the ChREBP pathway induced by glucose: *MLXIPL*^26^, *PKLR*^27^, *HCFC1*^28, 29^, *PPP2CA*^30^, and *BANF1*^31^.

**Supplementary Figure 1. Comparison of body fat distributions and anthropometric assessments strati**

**Supplementary Figure 1. Comparison of body fat distributions and anthropometric assessments stratified by the NAS between the HC and LC groups.** (A) The boxplots displaying adipose tissue amount measured using fat CT. visceral adipose tissue area; subcutaneous adipose tissue area; total adipose tissue area; sarcopenia rate obtained by division calculation of appendicular skeletal muscle mass (ASM) by BMI. (B) The boxplots showing anthropometric data. Total muscle mass; total fat mass; body weight; and waist circumference. Statistical analysis was performed with the Kruskal-Wallis test and Dunn’s multiple comparison test. * *P*<0.05, ***P*<0.01, ****P*<0.001.

A

**
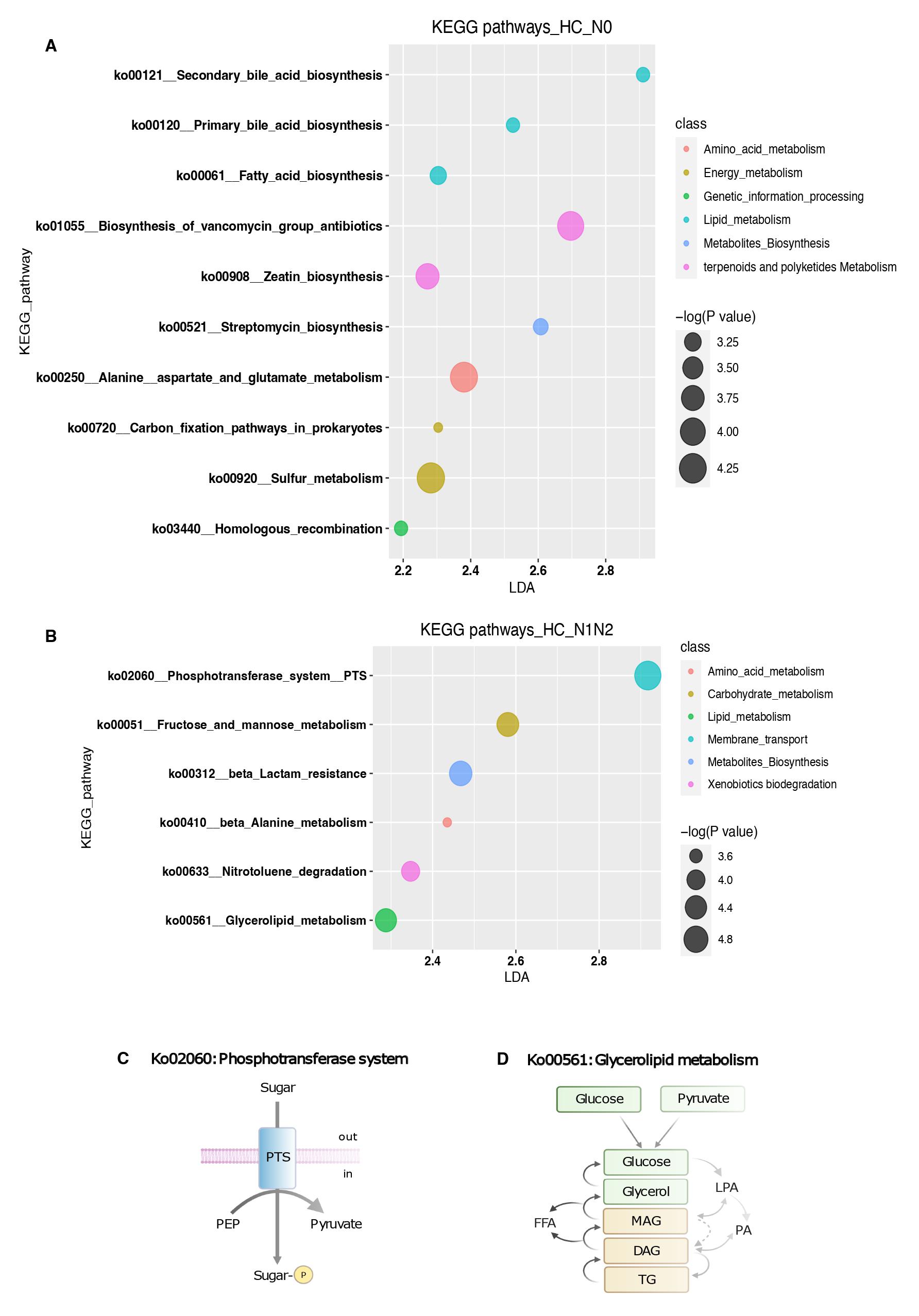
**

**Supplementary Figure 2. Microbial functional analysis prediction.** KEGG pathways significantly differed between non-NASH and NASH in the HC group, enriched in (A) N0 and (B) N1/N2 (LEfSe analysis, LDA score > 2, *P*<0.05). The bubble plot shows the KEGG pathways at the intersection of the PICRUSt2 findings. (C) Simplified scheme of significantly different KEGG pathways between non-NASH and NASH in the HC group displaying a phosphotransferase system and glycerolipid metabolism.

A

B

Supplementary Figure 3. Nutritional, clinical, and gut microbial characteristics of the twin cohort. (A) The pie charts displaying the percentage of energy intake from carbohydrate, fat, and protein in the HC and LC groups. (B) The heatmaps showing significant correlations between clinical variables and nutrition patterns (**P*<0.05, ***P*<0.01, ****P*<0.001). Positive correlations are expressed as red and negative correlations as blue.

**Abbreviations:** ALT, alanine transaminase; AST, aspartate transaminase; BMI, body mass index; FBG, fasting blood glucose; HDL, high-density lipoprotein; HOMA-IR, homeostasis model assessment of insulin resistance; hs-CRP, high-sensitivity C-reactive protein; LDL, low-density lipoprotein; tCholesterol, total cholesterol.

A

B

C

**Supplementary Figure 4. Comparison of the expression level of transcripts between the HC and LC groups.** The comparison of z-score transformed expression level of three transcripts between HC and LC are depicted in total population and each subgroup of NAFLD (N0, N1, and N2). (A) *SREBF2*, (B) *SIRT1,* and (C) *HCFC1*. Unpaired *t*-tests were used for statistical analysis. **P*<0.05

C

**C**

**Supplementary Figure 5. Relationship of clinical variables and gut microbes with transcripts.** (A) The heatmaps depicting significant correlations between clinical variables and transcriptomes in the HC and LC groups. (B) The heatmaps showing significant correlations between transcriptomes and gut microbes (**P*<0.05, ***P*<0.01, ****P*<0.001). Positive correlations are expressed as red and negative correlations as blue. (C) The linear regression models (with 95% CI band highlighted in grey) between transcriptomes and gut microbes after adjusted for BMI, age, and sex (red, HC; blue, LC).

**Abbreviations**: AST, aspartate transaminase; ALT, alanine transaminase; TB, total bilirubin; Plt, platelet; GGT, gamma-glutamyl transferase; C-peptide, connecting peptide; HbA1c, glycated hemoglobin; HOMA-IR, homeostasis model assessment of insulin resistance; LDL, low-density lipoprotein; Chol, cholesterol; TG, triglycerides; Alb, albumin; FFA, free fatty acids; hs-CRP, high-sensitivity C-reactive protein; HA, hyaluronic acid; adipo-IR, adipose tissue insulin resistance.

**Supplementary Table 1. Clinical characteristics of entire study subjects stratified by the NAFLD activity score**

**Abbreviations:** BMI, body mass index; AST, aspartate transaminase; ALT, alanine transaminase; GGT, gamma-glutamyl transferase; HDL, high-density lipoprotein; LDL, low-density lipoprotein; TG, triglycerides; FFA, free fatty acids; TB, total bilirubin; Alb, albumin; hs-CRP, high-sensitivity C-reactive protein; HA, hyaluronic acid; HbA1c, glycated hemoglobin; FBG, fasting blood glucose; Adipo-IR, adipose tissue insulin resistance; HOMA-IR, homeostasis model assessment of insulin resistance. Data are presented as the mean ± SD or n (%). Significant differences among subgroups are calculated using the Kruskal-Wallis test. **P*<0.05, ***P*<0.01, ****P*<0.001.

**Supplementary Table 2. Histological characteristics of entire study subjects stratified by carbohydrate dietary patterns and the NAFLD activity score**

Data are presented as the mean ± SD or n (%). Significant differences among subgroups are calculated using the Kruskal-Wallis test. **P*<0.05, ***P*<0.01, ****P*<0.001.

**Supplementary Table 3. Nutritional characteristics of study subjects stratified by carbohydrate dietary patterns and the NAFLD activity score**

Data are presented as the mean ± SD or n (%). Significant differences among subgroups are calculated using the Kruskal-Wallis test. **P*<0.05, ***P*<0.01, ****P*<0.001.

**Supplementary Table 4.** **Clinical and genetic characteristics of entire study subjects stratified by carbohydrate dietary patterns**

**Abbreviations:** BMI, body mass index; AST, aspartate transaminase; ALT, alanine transaminase; GGT, gamma-glutamyl transferase; HDL, high-density lipoprotein; LDL, low-density lipoprotein; TG, triglycerides; FFA, free fatty acids; TB, total bilirubin; Alb, albumin; hs-CRP, high-sensitivity C-reactive protein; HA, hyaluronic acid; HbA1c, glycated hemoglobin; FBG, fasting blood glucose; Adipo-IR, adipose tissue insulin resistance; HOMA-IR, homeostasis model assessment of insulin resistance; *PNPLA3*, patatin-like phospholipase domain-containing protein 3; *TM6SF2*, transmembrane 6 superfamily member 2; *SREBF2*, sterol regulatory element binding transcription factor 2; *MBOAT7*, membrane-bound O-acyltransferase domain containing 7; *HSD17B13,* 17β-hydroxysteroid dehydrogenase type 13. Data are presented as the mean ± SD or n (%). Significant differences among subgroups are calculated using the Kruskal-Wallis test. **P*<0.05, ***P*<0.01, ****P*<0.001.

**Supplementary Table 5. Nutritional and clinical characteristics of the twin cohort population stratified by hepatic steatosis index**

**Abbreviations:** FBG, fasting blood glucose; BMI, body mass index; AST, aspartate transaminase; ALT, alanine transaminase; GGT, gamma-glutamyl transferase; hs-CRP, high-sensitivity C-reactive protein; HOMA-IR, homeostasis model assessment of insulin resistance; tCholesterol, total cholesterol; HDL, high-density lipoprotein; LDL, low-density lipoprotein.

Data are presented as the mean ± SD or n (%). Significant differences among subgroups are calculated using the Kruskal-Wallis test. **P*<0.05, ***P*<0.01, ****P*<0.001.

**Supplementary Table 6. The distribution of genetic risk variants in the biopsy-proven NAFLD cohort.**

**Abbreviations:** *PNPLA3*, patatin-like phospholipase domain-containing protein 3; *TM6SF2*, transmembrane 6 superfamily member 2; *SREBF2*, sterol regulatory element binding transcription factor 2; *MBOAT7*, membrane-bound O-acyltransferase domain containing 7; *HSD17B13*, 17β-hydroxysteroid dehydrogenase type 13. Data are expressed as n (%). Significant differences among subgroups are calculated using the Kruskal-Wallis test. **P*<0.05, ***P*<0.01, ****P*<0.001.

**Supplementary Table 7. Glucose-induced and DNL-associated transcripts analysis**

**Abbreviations:** *GCK*, glucokinase; *G6PC*, glucose-6-phosphatase catalytic subunit 1; *PFKFB3*, 6-phosphofructo-2kinase/fructose 2,6-biphosphatase3; *PDHA1*, pyruvate dehydrogenase e1 subunit alpha1; *ACLY*, ATP citrate lyase; *ACACA*, Acetyl CoA carboxylase alpha; *ACACB*, acetyl-CoA carboxylase beta; *CREBBP*, CREB binding protein; *USF1*, upstream transcription factor1; *HDAC9*, histone deacetylase 9; *KAT2B*, lysine acetyltransferase 2B; *FASN*, fatty acid synthase; *FAS*, fas cell surface death receptor; *SCD*, stearoyl-CoA desaturase; *ELOVL6*, elongation of very long chain fatty acids protein 6; *PRKAA2*, 5-AMP activated protein kinase catalytic subunit alpha2; *SIRT1*, silent mating type information regulation 2 homolog 1; *SREBF1*, sterol regulatory element binding transcription factor 1; *SREBF2*, sterol regulatory element binding transcription factor 2; *PIK3CA*, phosphatidylinositol-4,5-bisphosphate 3-kinase catalytic subunit alpha; *AKT1*, AKT serine/threonine kinase 1; *PRKDC*, protein kinase, DNA-activated, catalytic subunit; *PRKCI*, protein kinase c iota ; *PRKCZ*, protein kinase c zeta type; *mTOR*, mechanistic target of rapamycin kinase; *LPIN1*, phosphatidate phosphatase-1; *IRS1*, Insulin receptor substrate 1; *GSK3B*, glycogen synthase kinase 3 beta; *TSC2*, TSC complex subunit 2; *INSIG2*, insulin induced gene 2; *FOXO1*, forkhead box o1; *NR1H3*, nuclear receptor subfamily 1 group h member 3; *PKLR*, pyruvate kinase L/R; *HCFC1*, host cell factor C1; *PPP2CA*, protein phosphatase 2 catalytic subunit alpha; *BANF1*, BAF nuclear assembly factor 1. Data are presented as the mean ± SD or n (%). Significant differences among subgroups are calculated using the Kruskal-Wallis test. **P*<0.05, ***P*<0.01, ****P*<0.001.

**Supplementary Table 8. Causal inference among gut microbial, clinical, and transcript factors in the HC and LC groups**

**Abbreviations:** AST, aspartate transaminase; ALT, alanine transaminase; HOMA-IR, homeostasis model assessment of insulin resistance; adipo-IR, adipose tissue insulin resistance; GGT, gamma-glutamyl transferase; FFA, free fatty acids; BMI, body mass index; *SIRT1*, Sirtuin 1; *mTOR*, mechanistic target of rapamycin kinase; *SREBF2*, sterol regulatory element binding transcription factor 2. Only significant causal correlations were included. (*P*<0.05) r*x|y represents for Pearson correlation coefficients direction from y to x. r*y|x represents opposite direction (from x to y). P value is the testing result of the significance of Pearson’s correlation coefficient (r).

**References**

1. Kolde R. Pheatmap: pretty heatmaps. R package version 2012; 1.

2. Team RC. R: A language and environment for statistical computing. 2013.

3. Love MI, Huber W, Anders S. Moderated estimation of fold change and dispersion for RNA-seq data with DESeq2. Genome Biol 2014; 15:550.

4. Wickham H. ggplot2. Wiley Interdisciplinary Reviews: Computational Statistics 2011; 3:180-5.

5. DeWaal D, Nogueira V, Terry AR, Patra KC, Jeon S-M, Guzman G, et al. Hexokinase-2 depletion inhibits glycolysis and induces oxidative phosphorylation in hepatocellular carcinoma and sensitizes to metformin. Nature Communications 2018; 9:446.

6. Nishikawa T, Bellance N, Damm A, Bing H, Zhu Z, Handa K, et al. A switch in the source of ATP production and a loss in capacity to perform glycolysis are hallmarks of hepatocyte failure in advance liver disease. Journal of Hepatology 2014; 60:1203-11.

7. Schoors S, De Bock K, Cantelmo Anna R, Georgiadou M, Ghesquière B, Cauwenberghs S, et al. Partial and Transient Reduction of Glycolysis by PFKFB3 Blockade Reduces Pathological Angiogenesis. Cell Metabolism 2014; 19:37-48.

8. Davidson Shawn M, Papagiannakopoulos T, Olenchock Benjamin A, Heyman Julia E, Keibler Mark A, Luengo A, et al. Environment Impacts the Metabolic Dependencies of Ras-Driven Non-Small Cell Lung Cancer. Cell Metabolism 2016; 23:517-28.

9. Zhao S, Jang C, Liu J, Uehara K, Gilbert M, Izzo L, et al. Dietary fructose feeds hepatic lipogenesis via microbiota-derived acetate. Nature 2020; 579:586-91.

10. Kim C-W, Addy C, Kusunoki J, Anderson NN, Deja S, Fu X, et al. Acetyl CoA Carboxylase Inhibition Reduces Hepatic Steatosis but Elevates Plasma Triglycerides in Mice and Humans: A Bedside to Bench Investigation. Cell Metabolism 2017; 26:394-406.e6.

11. Min AK, Jeong JY, Go Y, Choi YK, Kim YD, Lee IK, et al. cAMP response element binding protein H mediates fenofibrate-induced suppression of hepatic lipogenesis. Diabetologia 2013; 56:412-22.

12. Viscarra JA, Wang Y, Hong I-H, Sul HS. Transcriptional activation of lipogenesis by insulin requires phosphorylation of MED17 by CK2. Science Signaling 2017; 10:eaai8596.

13. Wong RHF, Chang I, Hudak CSS, Hyun S, Kwan H-Y, Sul HS. A Role of DNA-PK for the Metabolic Gene Regulation in Response to Insulin. Cell 2009; 136:1056-72.

14. Desert C, Baéza E, Aite M, Boutin M, Le Cam A, Montfort J, et al. Multi-tissue transcriptomic study reveals the main role of liver in the chicken adaptive response to a switch in dietary energy source through the transcriptional regulation of lipogenesis. BMC Genomics 2018; 19:187.

15. Chong MF-F, Hodson L, Bickerton AS, Roberts R, Neville M, Karpe F, et al. Parallel activation of de novo lipogenesis and stearoyl-CoA desaturase activity after 3 d of high-carbohydrate feeding. The American Journal of Clinical Nutrition 2008; 87:817-23.

16. Strable MS, Ntambi JM. Genetic control of de novo lipogenesis: role in diet-induced obesity. Critical Reviews in Biochemistry and Molecular Biology 2010; 45:199-214.

17. Ganbold M, Ferdousi F, Arimura T, Tominaga K, Isoda H. New Amphiphilic Squalene Derivative Improves Metabolism of Adipocytes Differentiated From Diabetic Adipose-Derived Stem Cells and Prevents Excessive Lipogenesis. Front Cell Dev Biol 2020; 8:577259-.

18. Tobita T, Guzman-Lepe J, Takeishi K, Nakao T, Wang Y, Meng F, et al. SIRT1 Disruption in Human Fetal Hepatocytes Leads to Increased Accumulation of Glucose and Lipids. PLOS ONE 2016; 11:e0149344.

19. Yamashita T, Honda M, Takatori H, Nishino R, Minato H, Takamura H, et al. Activation of lipogenic pathway correlates with cell proliferation and poor prognosis in hepatocellular carcinoma. Journal of Hepatology 2009; 50:100-10.

20. Kim JY, Garcia-Carbonell R, Yamachika S, Zhao P, Dhar D, Loomba R, et al. ER Stress Drives Lipogenesis and Steatohepatitis via Caspase-2 Activation of S1P. Cell 2018; 175:133-45.e15.

21. Li X, Tao J, Cigliano A, Sini M, Calderaro J, Azoulay D, et al. Co-activation of PIK3CA and Yap promotes development of hepatocellular and cholangiocellular tumors in mouse and human liver. Oncotarget 2015; 6:10102-15.

22. Calvisi DF, Wang C, Ho C, Ladu S, Lee SA, Mattu S, et al. Increased Lipogenesis, Induced by AKT-mTORC1-RPS6 Signaling, Promotes Development of Human Hepatocellular Carcinoma. Gastroenterology 2011; 140:1071-83.e5.

23. Shao W, Espenshade Peter J. Expanding Roles for SREBP in Metabolism. Cell Metabolism 2012; 16:414-9.

24. Deng X, Zhang W, O-Sullivan I, Williams JB, Dong Q, Park EA, et al. FoxO1 Inhibits Sterol Regulatory Element-binding Protein-1c (SREBP-1c) Gene Expression via Transcription Factors Sp1 and SREBP-1c*. Journal of Biological Chemistry 2012; 287:20132-43.

25. Krycer JR, Brown AJ. Cross-talk between the Androgen Receptor and the Liver X Receptor: IMPLICATIONS FOR CHOLESTEROL HOMEOSTASISThe on-line version of this article (available at <http://www.jbc.org>) contains supplemental Table S1. Journal of Biological Chemistry 2011; 286:20637-47.

26. Herman MA, Peroni OD, Villoria J, Schön MR, Abumrad NA, Blüher M, et al. A novel ChREBP isoform in adipose tissue regulates systemic glucose metabolism. Nature 2012; 484:333-8.

27. Sae-Lee C, Moolsuwan K, Chan L, Poungvarin N. ChREBP Regulates Itself and Metabolic Genes Implicated in Lipid Accumulation in β–Cell Line. PLOS ONE 2016; 11:e0147411.

28. Vella P, Scelfo A, Jammula S, Chiacchiera F, Williams K, Cuomo A, et al. Tet Proteins Connect the O-Linked N-acetylglucosamine Transferase Ogt to Chromatin in Embryonic Stem Cells. Molecular Cell 2013; 49:645-56.

29. Ido-Kitamura Y, Sasaki T, Kobayashi M, Kim H-J, Lee Y-S, Kikuchi O, et al. Hepatic FoxO1 Integrates Glucose Utilization and Lipid Synthesis through Regulation of Chrebp O-Glycosylation. PLOS ONE 2012; 7:e47231.

30. Uyeda K, Yamashita H, Kawaguchi T. Carbohydrate responsive element-binding protein (ChREBP): a key regulator of glucose metabolism and fat storage. Biochemical pharmacology 2002; 63:2075-80.

31. Puig-Oliveras A, Ramayo-Caldas Y, Corominas J, Estellé J, Pérez-Montarelo D, Hudson NJ, et al. Differences in muscle transcriptome among pigs phenotypically extreme for fatty acid composition. PloS one 2014; 9:e99720.
